# In situ differences in nitrogen cycling related to presence of submerged aquatic vegetation in a Gulf of Mexico estuary

**DOI:** 10.1002/ecs2.4290

**Published:** 2022-12-04

**Authors:** R. S. Fulford, K. Houghton, J. James, M. Russell

**Affiliations:** 1 Office of Research and Development, US, Environmental Protection Agency, Gulf, Breeze, Florida, USA; 2 Centers for Disease Control and Prevention, Atlanta, Georgia, USA

**Keywords:** denitrification, estuary, habitat management, nitrogen fixation, SAV

## Abstract

Estuaries provide a suite of ecosystem services to people but are also under heavy stress from human development including excess nutrient loading and alterations in benthic habitat that affect nutrient cycling. Here we examine the interaction of two important and common ecosystem management priorities in estuaries: limiting eutrophication and restoration of submerged aquatic vegetation (SAV). Rates of benthic nitrogen processing can vary by habitat type and there is need for more complete data on the contribution of SAV to overall nitrogen cycling in estuaries, as well as a need to examine nitrogen cycling in situ to better characterize the role of SAV areal coverage in mediating estuarine eutrophication. We compare nitrogen cycling between two common and adjacent habitat types (SAV and adjacent bare sediment [BS]) in an index coastal estuary using an in situ chamber-based approach to better capture realized habitat differences. We also examined genomic community structure of sediment bacteria and archaea to identify biological indicators of nitrogen exchange. Both mean sediment–water exchange of dissolved N_2_ and microbial functional community structure differed between SAV and BS. Habitat differences were more consistent with lower variability at locations with low salinity and when sediment organic content was highest, which aligns with findings in other studies. Habitat types differed significantly in microbial composition, including functional groups and genes, like *nifH*, that may contribute to observed differences in nitrogen cycling. Overall, habitat type appeared most important to nitrogen cycling near the river mouth where sediment nitrogen was higher, and this information has implications for integrated management of habitat restoration/conservation and nutrient loading.

## INTRODUCTION

Estuaries are complex and important ecosystems that are under heavy stress from human development. Eutrophication of estuaries is a particular concern because of both anthropogenic nutrient loading (Caffrey & Murrell, 2016) and alterations in benthic habitat that affect nutrient cycling (La Peyre et al., 2014; Piehler & Smyth, 2011). Natural processing of anthropogenic nitrogen, particularly at the sediment–water interface, is an important estuarine ecosystem service that is impacted by shifts in benthic habitat (Eyre et al., 2016). Estuarine benthic habitat is dynamic and complex and understanding how nitrogen cycling contributes to eutrophication requires that we understand how nitrogen cycling differs among different benthic habitat types. Particularly important are those habitat types most likely to change in response to human influence. A key example is estuarine submerged aquatic vegetation (SAV). Understanding differences and patterns in nitrogen recycling/removal between SAV and adjacent bare sediment (BS) is necessary to assess the relative role of increasing/decreasing SAV coverage on the ecosystem service of nitrogen processing. A lack of consensus in habitat-specific nitrogen exchange rates complicates the use of these data for decision-making. Here we examine the interaction of two important and common priorities in estuaries: limiting anthropogenic nitrogen concentration and restoration of SAV. The work focuses on the ecosystem services of SAV by measuring changes in nitrogen processing rates between SAV and adjacent BS, which could potentially become SAV through restoration. We address the question of how SAV restoration may be evaluated by its contribution to the ecosystem service of natural processing of nitrogen at the scale of the whole estuary to inform habitat management decisions and nitrogen loading criteria.

Rates of benthic nitrogen processing vary greatly by bottom type (Eyre & Maher, 2011; Hamersley & Howes, 2005; Smyth et al., 2015). SAV habitat is highly productive and typically hosts a high rate of nitrogen cycling supported by availability of organic matter and tight spatial coupling between nitrification and denitrification (Devereux et al., 2011; Eyre et al., 2013). Rates of denitrification have been reported as higher in SAV (Eyre et al., 2013; Garcias-Bonet et al., 2018) but have also been reported as lower in SAV compared with adjacent BS (Ottosen et al., 1999; Risgaard-Petersen et al., 1998). Nitrogen fixation has also been reported as both an important (Carlson-Perret et al., 2019; Cole & McGlathery, 2012) and negligible (Hoffman et al., 2019) process in SAV. The role of SAV in nitrogen cycling and sequestration at the scale of estuaries is also difficult to estimate because most work on nitrogen cycling in SAV has been done at a very small scale (in centimeters) to avoid bias generated by leaf and root damage that is typical for ex situ analysis (e.g., Hoffman et al., 2019; Piehler & Smyth, 2011). Estuarine SAV coverage is typically heterogeneous at this scale, complicating the process of scaling up to larger, management-relevant scales. Yet, efforts to conduct analysis of nitrogen cycling at more relevant scales have borne fruit. For instance, a comprehensive examination of nitrogen processing at the scale of estuaries in Australia revealed that SAV had a much higher than anticipated influence on whole-system nitrogen processing (Eyre et al., 2016). Efforts at functionally mapping habitat at the estuary scale have demonstrated the value of understanding the functional impacts of habitat change (Eyre & Maher, 2011). There is a need for more complete data on the role of SAV in nitrogen cycling in estuaries compared with other bottom types, as well as on the drivers of these habitat differences, to better characterize how the investment in SAV areal coverage may help slow estuarine eutrophication.

The role of different bottom types in processing nitrogen at the scale of the estuary is also mediated by differences in the microbial community responsible for nitrogen processing (Fulweiler et al., 2013; Hoffman et al., 2019). Variability in dominant nitrogen processing pathways can be mediated by differences in microbial community composition or differences in microbial response to nitrogen species availability (Hoffman et al., 2019). Detectable differences in nitrogen processing can be better ascribed to habitat differences if they are associated with differences in microbial community composition, and this association is important to properly understand habitat-specific effects (Devereux, 2005; Ettinger et al., 2017). Here we focus on both microbial community differences and functional differences in targeted N-cycling genes as a method of understanding functional differences between habitat types.

Examination of habitat effects on nitrogen cycling at the estuarine scale requires information on the differences in functional rates among adjacent benthic habitat types so that transitions between habitat types (e.g., BS to SAV) can be evaluated based on estuarine functional response. Information about how benthic nitrogen cycling differs among adjacent bottom types will aid in our understanding of how estuaries respond functionally to nutrient loading and how that response changes as SAV coverage changes. Data on habitat differences in functional rates that can be scaled up to the entire estuary will greatly aid management planning for whole systems. The objective here is to test the hypotheses that nitrogen cycling rates, nitrogen processing microbial community, and organic content of the sediment differ between two common and adjacent bottom types (SAV and BS) in an index coastal estuary. The goal is to better understand the functional equivalency of these important habitat types for processing of anthropogenic nitrogen for information management.

## METHODS

### Site description

**FIGURE 1:**
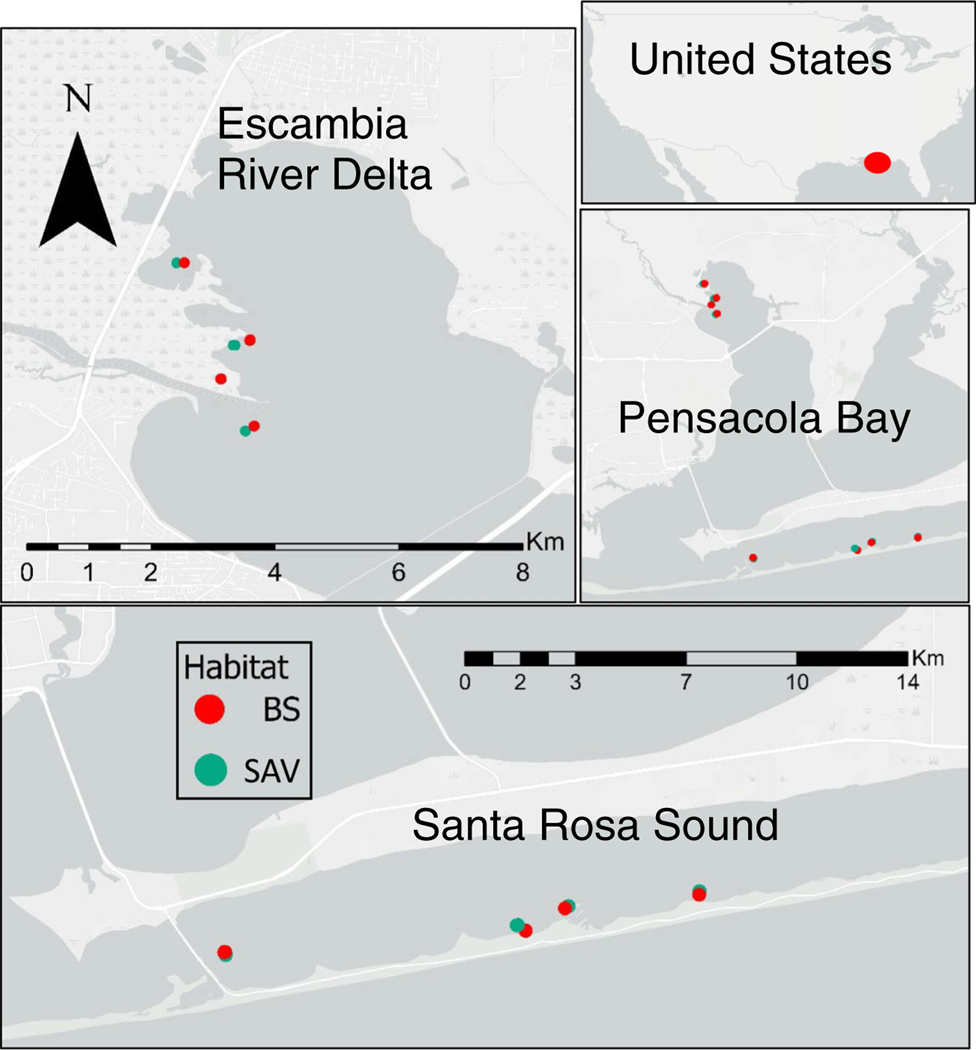
Map showing two sample locations in Pensacola Bay System, FL, USA. Locations are Escambia River delta (upper left) and Santa Rosa Sound (lower). Randomly selected sample sites for each location are indicated by paired green (submerged aquatic vegetation [SAV]) and red (bare sediment [BS]) dots. See text for details.

The Pensacola Bay System (PBS) is a river-dominated estuary on the north-central coast of the Gulf of Mexico (Figure 1) characterized by multiple benthic habitat types, including historically extensive SAV (Lewis et al., 2008). The PBS is a shallow (<3 m mean depth), micro-tidal system (<0.5 m range) with a high water-residence time (>18 days), making it sensitive to excess nitrogen loading and eutrophication. Coverage of SAV has declined more than 95% since its peak in the 1950s but has shown recent signs of recovery in parts of the Pensacola Bay estuary (Lewis et al., 2016). This recovery has been contemporaneous with a decline in the anthropogenic nitrogen load into the Bay.

This study was conducted at the two major areas within the PBS known to support SAV habitat (Figure 1). The mouth of the Escambia River (ER) is at the northern boundary of Pensacola Bay proper and is characterized by low salinity, higher levels of sediment organic matter, and higher likelihood of hypoxic conditions in the summer. The dominant SAV species at this location are *Vallisneria* spp. mixed with *Ruppia* spp. (Table 1). Santa Rosa Sound (SR) is in the southern estuary portion of PBS just adjacent to the Gulf of Mexico. The SR location is characterized by higher salinity, higher water clarity, low-sediment organic matter, and normoxic conditions typical in all seasons of the year (Table 1). The dominant SAV species in SR are *Thalassia* spp. and *Halodule* spp. Mean depth in both locations is approximately 2 m. Sampling occurred on four dates within each location, two per season per year. Our goal was to examine habitat differences within each location (river mouth/lower estuary) not a comparison between locations so to focus on differences at a spatial scale relevant to restoration, but to do so in both potential locations for SAV restoration.

### Nitrogen rate estimates from chambers

**FIGURE 2:**
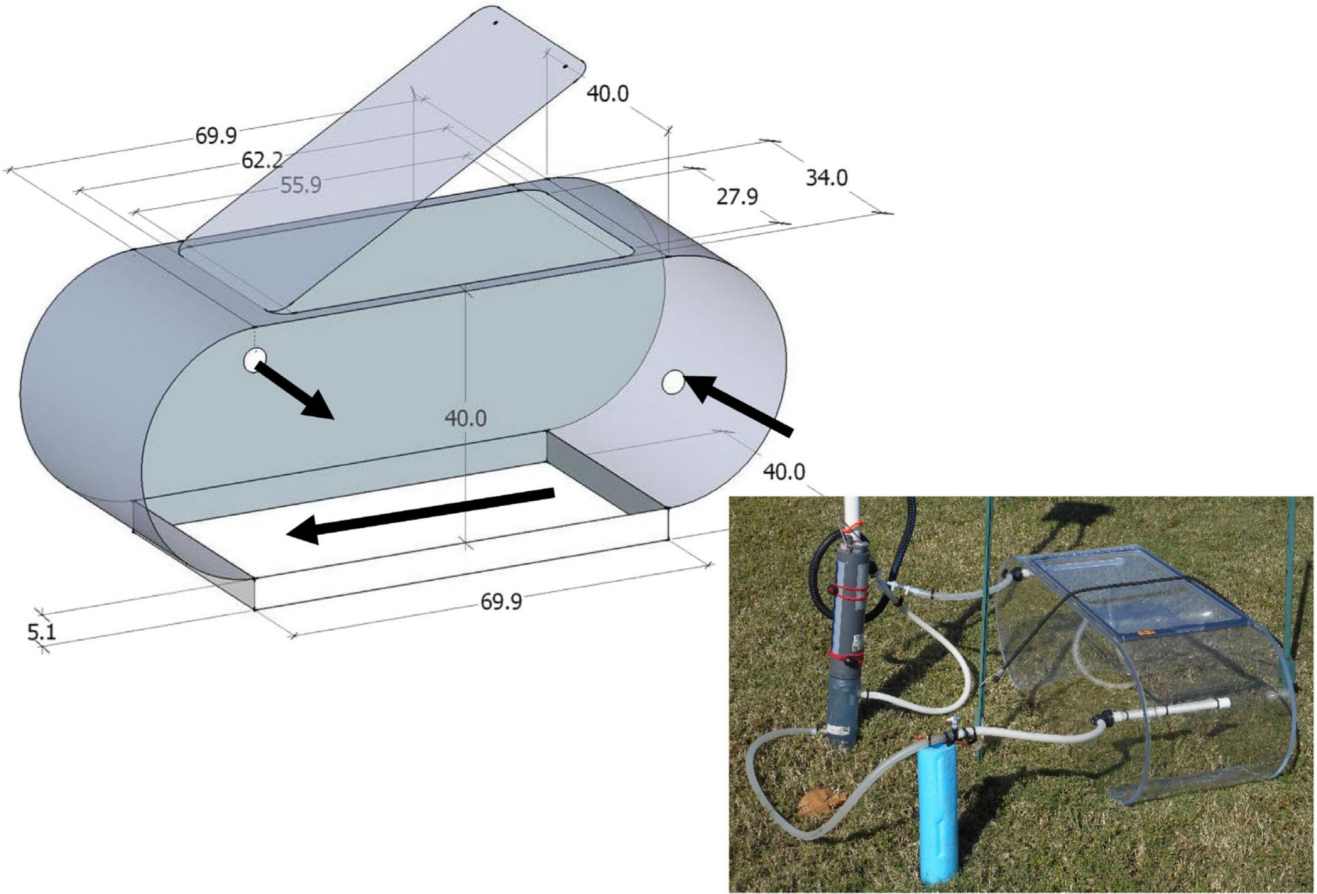
Details and dimensions (in centimeters) of benthic chamber design intended to facilitate linear flow over the sediment surface. The design was adapted from Webb and Eyre (2004) but increased in size with an equivalent surface/volume ratio to their chamber. The lower picture shows setup for deployment including water circulating system with inline water quality multiprobe, and sample port.

Habitat-specific rates of nitrogen exchange at the sediment–water interface were estimated with in situ chamber incubations in SAV and adjacent BS. The chambers were used to avoid bias created by smaller surface area core incubations in vegetated sediment (Eyre et al., 2011). The chambers (volume: 163 L; surface area: 0.28 m^2^) were designed to maintain both SAV shape and natural flow dynamics over the sediment surface (Webb & Eyre, 2004). Water flow over the sediment should be laminar rather than circular, which is common in rectangular or cylindrical chambers and about 1 cm s^−1^ for this system based on previous observations. Natural flow is maintained through a combination of chamber shape (Figure 2), chamber orientation in the water column, and directional flow created using a spray bar inside the chamber (Figure 2). In addition, the sediment surface area was higher than that reported for core studies to measure a more averaged exchange rate. The chambers were deployed during both daylight and night hours in two habitat types (SAV and adjacent BS) at two locations in the study system (SR and ER; Table 1, Figure 1). Both locations have known natural beds of SAV, but SR has highly permeable sandy sediment and ER has less permeable sediment dominated by mud/silt. Sampling was conducted four times at each location/habitat between July 2015 and October 2017. Sampling times were evenly divided between summer (July/August) and fall (October/November) seasons.

The incubation chambers used were rectangular but with rounded ends to facilitate laminar water flow inside the chamber (Figure 2). Flow was maintained with a pump system that removed water from the right side of the chamber near the mid depth and reintroduced it to the chamber on the opposite side near the top. Nozzles were used to direct inflow along the rounded end and across the bottom of the chamber to generate flow across the sediment surface. The flow system was also used for monitoring water quality (primarily dissolved oxygen [DO]) and collecting water samples during incubations.

Sampling was conducted sequentially at each location with regard to habitat type and day/night sampling. Daytime sampling was planned to closely monitor and eliminate air bubbles inside the chambers that could bias nitrogen concentration estimates (Eyre et al., 2011). Preliminary testing showed that air bubble bias could be prevented by presoaking chambers and all tubing for 24 h prior to deployment and by scheduling daytime incubations to begin prior to dawn to provide a reference point for potential bias. Oxygen saturation inside the chambers was monitored during incubations and not allowed to exceed 80% or drop below 20%, and chambers were visually inspected at each sample time point for presence of air bubbles. If air bubbles were observed, the trial was ended at the previous time point. If internal DO concentration dropped below 20%, the replicate was discarded. Chambers were randomly deployed in triplicate at each location/habitat type at least 6 h before the start of an incubation. Over the entire course of sampling, one replicate was discarded based on the presence of air bubbles inside the chamber, no replicates were discarded based on minimum DO levels.

Each chamber was placed randomly within location and habitat type and pushed into the sediment so that the lower edge inside the chamber was even with the sediment surface. This allowed the collar of the chamber to penetrate the sediment to a depth of 10.2 cm, creating a tight seal between the inside and outside. Preliminary testing with dye demonstrated that once the chambers are seated in the sediment, they are sealed to prevent unwanted exchange of water with the surrounding environment. Once a chamber was in place, the flow system was assembled, and the chambers (3) were left open with circulating water until the start of the incubation period.

The incubation period was initiated either 2 h before dawn (day) or 1 h after full dusk (night). The day sampling period included predawn data as a reference point, but all data used in measuring daytime exchange rates were collected after full sunrise. At time zero, the chamber lid was closed and sealed, and the first water sample was collected. Water samples were collected in triplicate with a syringe attached to a sample valve located in the external flow line (Figure 2). Water samples for estimation of dinitrogen (N_2_) gas were collected by filling a glass vial (7 ml) to overflowing. The sample was treated with 10 μl 10% NaOH solution to fix the sample and then sealed with a glass stopper. These samples were stored submerged in water until analyzed. Water samples for NH_4_^+^ and NO concentrations were filtered with a GF/F filter and stored on ice for transportation to the lab where samples were stored until analyzed at 4°C and −20°C for NH_4_^+^ and NO_x_ samples, respectively. Samples were collected each hour for 6 h. DO concentration, temperature, and salinity were monitored inside each chamber during each incubation with a water quality probe (Manta Corporation) located in the external flow system. At the end of the incubation period, triplicate core samples (diameter of 2 cm) were collected to a depth of 2 cm from the inside and outside of each chamber. Most of the cores were stored at 4°C for sediment analysis, but three cores from each trial were frozen on dry ice and stored at −70°C for characterization of bacterial and archaeal community genomic composition.

### Analytical chemistry

Water samples were returned to the lab and stored for analysis. Concentrations of N_2_ gas were estimated with multi-inlet mass spectrometry (MIMS; Kana et al., 1994) based on a N_2_/Ar ratio approach. The N_2_ water samples were calibrated with N_2_-saturated Mill-Q water at the same temperature with rechecks of standard concentration every 15 samples. Concentrations of NH_4_^+^ were estimated with a fluorometer based on an analysis-specific standard curve with an *R*^2^ value >0.99 and rechecks of standard concentrations every 15 samples (Holmes et al., 1999). Concentrations of NO_x_ were estimated based on standard colorimetric approaches with a continuous flow analyzer. Concentrations of NO_x_ were based on an analysis-specific standard curve with an *R*^2^ value >0.99 and rechecks of standard concentrations every 15 samples. The established method detection limit for this analysis was 0.11 μM based on independent analysis of standards. All chemistry was conducted based on US Environmental Protection Agency (EPA) standard methods including periodic rechecks of laboratory blanks and sample replicates for consistency (APHA, 1998; Holmes et al., 1999).

### Sediment analysis

Sediment collected from each chamber deployment was dried to a constant weight at 60°C for estimation of sediment dry weight and combusted at 500°C to a constant weight for estimation of sediment organic content based on loss on ignition. Subsamples of sediment dried at 60°C (approximately 20 mg) were ground to a fine powder and analyzed for total particulate carbon and nitrogen with a CE Elantech Flash EA elemental analyzer (https://www.ceelantech.com/).

### Genomic composition

Sediment samples were analyzed for overall microbial genomic community structure at the family and genus level to differentiate the relative abundance of nitrogen processors among habitat types. Microbial DNA was extracted from sediment samples using the “MagAttract PowerSoil DNA KF kit” (Qiagen, Hilden, Germany) optimized for the KingFisher Duo platform (ThermoFisher Scientific, Waltham, MA, USA). Partial 16S ribosomal RNA (rRNA) gene fragments were amplified using the forward primer 515fB and the reverse primer 806rB targeting the Variable 4 (V4) region of the small subunit 16S rRNA in bacteria and archaea (Apprill et al., 2015; Caporaso et al., 2012). Paired-end (251 × 251 bp) MiSeq sequences were sequenced using the Caporaso et al. (2012) sequencing methods along with a mock community composed of 20 known bacterial strains (Microbial Mock Community B, HM-7983D; BEI Resources, National Institute of Allergy and Infectious Disease, National Institutes of Health, as part of the Human Microbiome Project).

### Quantitative polymerase chain reaction amplification

Quantitative polymerase chain reaction (qPCR) was performed to quantify nitrogen cycling genes in DNA extracted from sediment samples at Argonne National Laboratory using the Roche Light Cycler (lifescience.roche.com) and following the Minimum Information for Publication of Quantitative realtime PCR Experiments (MIQE) guidelines (Bustin et al., 2009). The qPCRs were performed using SYBR Green PCR Master Mix (Applied Biosystems, Foster City, CA, USA). Primers were selected (Table 2) targeting nitrogen cycling functional genes (Smith et al., 2017) *amoA* (archaea) (Meinhardt et al., 2015), *amoA* (bacteria) (Meinhardt et al., 2015), *nifH* (Mohan et al., 2004; Welsh et al., 2014), *nirS* (Kandeler et al., 2006), and *nosZ*(I) (Henry et al., 2006). The 16S rRNA gene (Apprill et al., 2015; Parada et al., 2016) qPCR conditions were optimized for each gene using gBlocks (Table 3; Integrated DNA Technologies, Coralville, IA, USA), starting with conditions in the original manuscripts for each primer pair. A consensus standard curve was generated for each set of primers, using the data from all four plates run for each gene. The Ct value for each well was recorded and the consensus curve was used to quantify copies per reaction. Since the template DNA for each sample was extracted from a known amount of sediment, all samples were calculated as copies per milligram of sediment. The copies of 16S rRNA per milligram of sediment were used to normalize all samples to a ratio of functional gene/16S rRNA.

### Data analysis

Exchange of nitrogen at the sediment–water interface was estimated with linear regression for change in water column concentration over time. The slopes (in micro-moles per minute) for concentration of N_2_, NO_x_, or NH_4_^+^ as a function of sample time point were calculated independently for three replicate chambers for each date, diel period, and habitat type at each location (estuary and river mouth). Slopes were tested for linearity by comparative fitting of the data, weighted by independent *R*^2^ values, and averaged across replicates. The mean weighted slope values were then compared by season, diel period, and habitat type in an ANOVA in R (R Core Team, 2017) using the “lm” package. The samples were considered independent as the sample locations (ER/SR) are large and highly heterogeneous in SAV coverage and sample sites were randomly selected each time we collected data (Figure 1). A diel nitrogen budget was developed for each habitat and season based on the *R*^2^-weighted mean exchange rates and estimates of Julian day length within season. Analysis of oxygen exchange rates proceeded in a similar fashion, except that no diel budget was developed for oxygen exchange. Sediment organic content (ash free dry weight) was also compared across habitat types within location with an ANOVA as a measure of habitat differences.

Amplicon data were analyzed with mothur version 1.39.5 (Kozich et al., 2013) using the Silva version 132 database (Pruesse et al., 2007; Quast et al., 2013). The 16S rRNA gene sequence amplicons were assembled into contigs from paired-end reads, and any contigs with ambiguous base pairs were discarded, as were any contigs over 260 bp. Contigs were aligned to the V4 region of the 16S rRNA Silva database (accessed 5 October 2018). Chimeras were screened using vsearch (Rognes et al., 2016), and contigs were classified with the Silva taxonomy database. Contigs classified as chloroplast, mitochondria, eukaryote, or unknown were removed from the database. The remaining contigs were clustered into operational taxonomic units (OTUs) employing a dissimilarity threshold of 0.03. All samples were subsampled to 10,933 sequences per sample.

Genomic composition data based on analysis of 16S rRNA genes for sediment bacteria and archaea were examined for pattern across habitat types within location based on a similarity analysis of identifiable OTUs. Sequence data were examined for consistency and to remove unknown and low incidence sequences before analysis. Removal of sequences of OTUs that represented less than 1% of the total number of observed sequences was determined as a cutoff that would not bias the data analysis based on a permutation test (Clarke & Warwick, 2001). The filtered dataset was then analyzed for similarity with a hierarchical agglomerative cluster analysis based on a Bray–Curtis resemblance matrix and an analysis of similarity (ANOSIM) (PRIMER-e, www.primer-e.com). These results were then plotted by habitat type within location for comparison based on multidimensional scaling (MDS). Finally, the Bray–Curtis matrix was used for SIMPER analysis to identify influential OTUs.

Copy numbers for the 16S rRNA gene, archaeal *amoA*, bacterial *amoB*, *nirS*, *nifH*, and *nosZ* were calculated per gram of sediment. For each sample, the functional gene copy number was divided by the copy number of the 16S rRNA gene. When comparing all the nitrogen cycling genes, the ratios were log(*x* + 1) transformed and standardized. When sample resemblances were based on individual functional genes, the ratios were not transformed. Copy number variability was analyzed using Plymouth Routines in Multivariate Ecological Research version 7.0.17 (Quest Research Limited, Albany, Auckland, New Zealand). Bray–Curtis similarity matrices were generated for sample resemblances based on functional gene copy number (Clarke et al., 2006). Resemblance matrices based on nitrogen cycling genes were compared with the resemblance matrix based on the 16S rRNA gene using RELATE.

Variance was analyzed using permutational multivariate analysis of variance (PERMANOVA+) (Type I [sequential] sums of squares, 9999 permutations, Monte Carlo tests) (Anderson, 2017).

## RESULTS

### Nitrogen cycling

**FIGURE 3:**
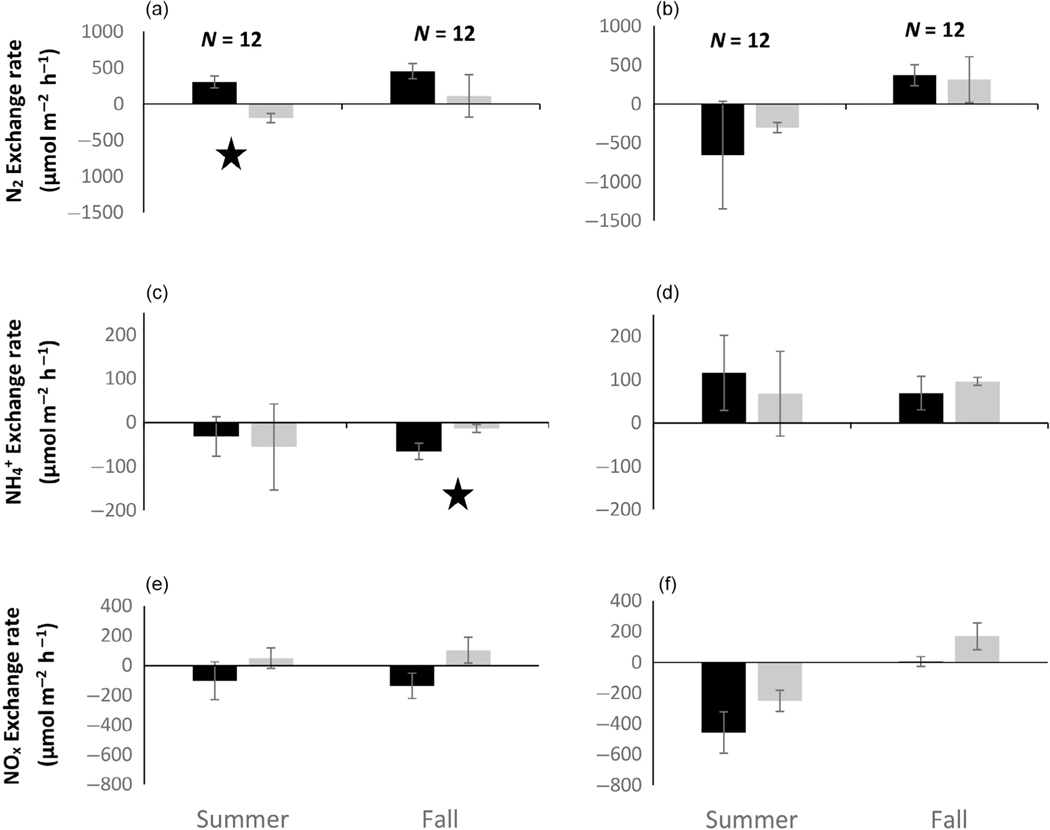
Mean (SD) net exchange in dissolved N_2_ (a, b), NH_4_^+^ (c, d), and NO_x_ (e, f) in the water column observed in in situ chamber incubations at the Escambia River mouth sample location. Data are separated by day (a, c, e) and night (b, d, f) sample periods. Bars within each figure are separated by season and habitat type: submerged aquatic vegetation (gray) and bare sediment (black). Sample size (*N*) is given by trial and indicates the total number of replicate chambers across habitat types. Significant differences between habitat types are indicated with a star. See text for further details.

**FIGURE 4:**
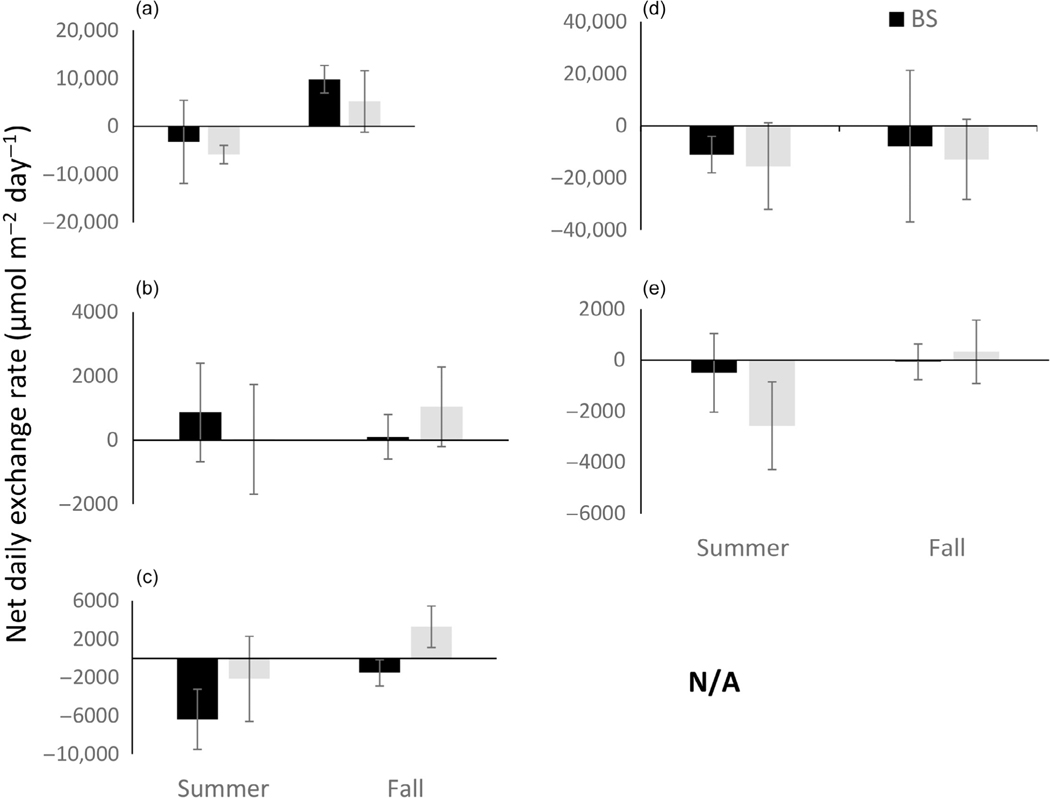
Net 24-h exchange budget for N_2_ (a, d), NH_4_^+^ (b, e), and NO_x_ (c) for the Escambia River (a–c) and Santa Rosa Sound (SR; d, e) sample locations. Bars within each figure are separated by season and habitat type: submerged aquatic vegetation (SAV; gray) and bare sediment (BS; black). Incubations were 4–6 h in length and each bar represents 6–12 chamber replicates. See text for further details. A budget for NO_x_ in SR was not calculated as most samples were <minimum detectiuon limit (MDL) for this analyte at this location.

Rates of exchange in nitrogen constituents were different between SAV and adjacent BS and between night and day samples (Figures 3 and 4), and the pattern of habitat differences differed between locations. Dissolved N_2_ exchange (slope) differed significantly between habitat types (*F*_1,95_ = 4.97, *p* = 0.03) and location by habitat type interaction (*F*_4,95_ = 4.74, *p* = 0.0019). There were also observed differences between locations (ANOVA *F*_1,95_ = 6.61, *p* = 0.015) but these were expected based on differences in salinity, SAV species, sediment organic content, and DO (Table 1).

At the river mouth location (ER), mean exchange of dissolved N_2_ between sediment and water column differed more between habitat types (SAV and BS) during the day than at night with a net positive value in BS and a zero or net negative value in SAV (Figure 3a,b). Daytime differences in N_2_ exchange were only significant in the summer. At night, differences in N_2_ exchange were near zero in both seasons and habitat types. When diel hourly data were combined to estimate a net daily N_2_ exchange budget for each habitat type, the result suggested that net daily N_2_ exchange did not differ by habitat type at the freshwater riverine location within either season but did differ between seasons with net negative (uptake) in the summer and net positive (efflux) in the fall (Figure 4a).

Exchange of inorganic N differed between locations and seasons, but less so across habitat types. Ammonium exchange between sediment and water column was significantly different between locations (*F*_1,95_ = 9.92, *p* = 0.002), but not between habitat types (*F*_1,95_ = 1.51, *p* = 0.22). There was significant interaction between location, habitat, and diel period (*F*_4,95_ = 3.13, *p* = 0.019), as well as changes in the spatial patterns between day and night sampling periods. At the freshwater location (ER), differences in exchange rates of ammonium were only observed between habitat types in the fall during the day, with larger uptake of ammonium in BS compared with SAV (Figure 3c,d). The exchange of NO_x_ at the freshwater location was more complicated with some habitat differences evident during the day; however, they were not significant (Figure 3e; *p* > 0.05). At night, NO_x_ exchange rates did not indicate habitat differences but did indicate seasonal differences with net uptake in the summer and net efflux (SAV) or zero exchange (BS) in the fall (Figure 3f; *p* = 0.03). The net daily exchange rates for inorganic N at the freshwater location were characterized by high variability and near neutral exchange over the diel period. Only NO_x_ in BS in the summer showed a net negative exchange rate (Figure 4b,c) over the diel period.

**FIGURE 5:**
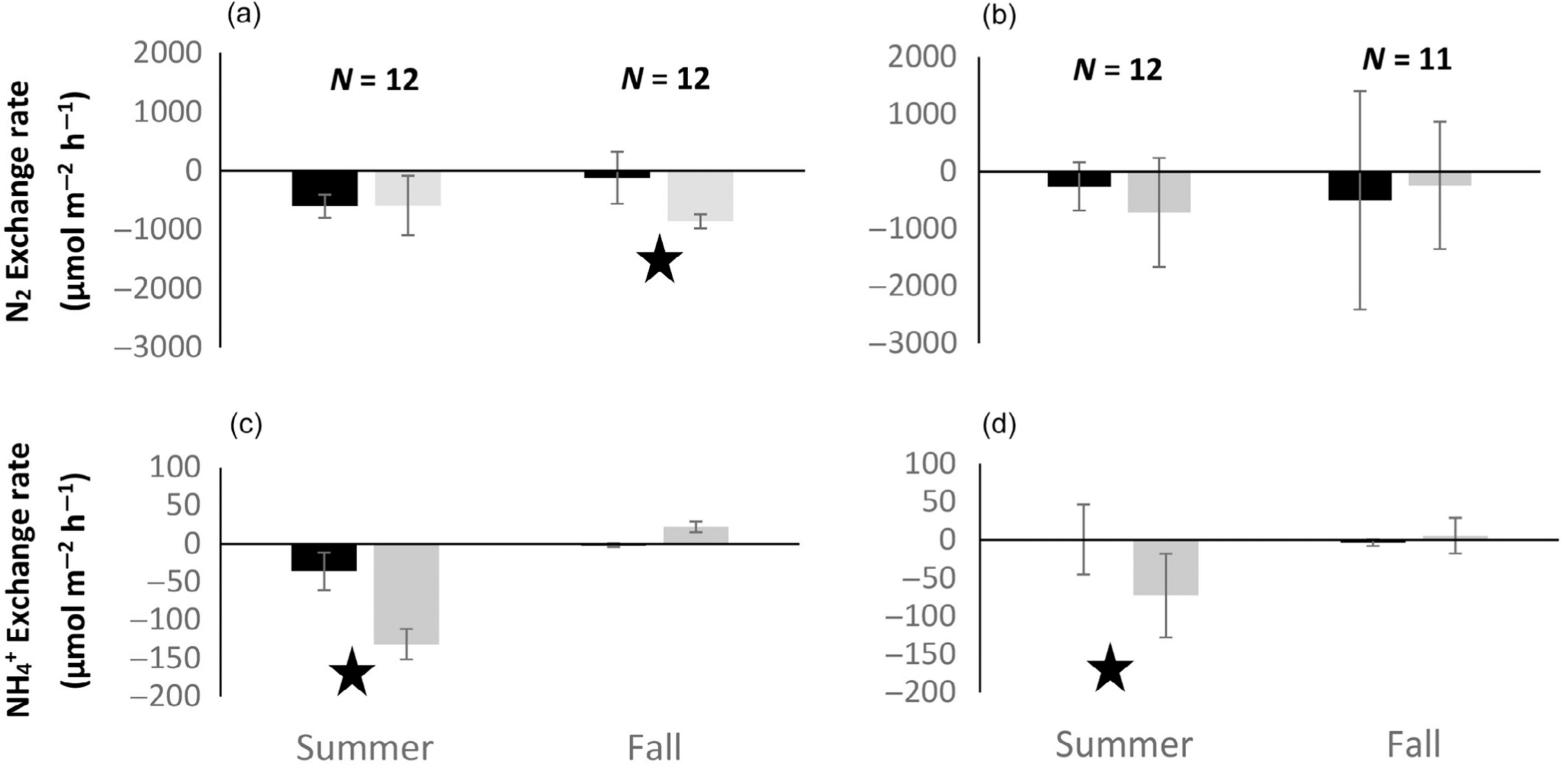
Mean (SD) net exchange in dissolved N_2_ (a, b) and NH_4_^+^ (c, d) in the water column observed in in situ chamber incubations at the Santa Rosa Sound (SR) mouth sample location. Data are separated by day (a, c) and night (b, d) sample periods. Bars within each figure are separated by season and habitat type: submerged aquatic vegetation (gray) and bare sediment (black). Sample size (*N*) is given by trial and indicates the total number of replicate chambers across habitat types. Significant differences between habitat types are indicated with a star. Rate data for NO_x_ in SR were not calculated as most samples were <minimum detectiuon limit (MDL) for this analyte at this location. See text for further details.

At the lower estuary location (SR), N_2_ flux was generally negative (uptake) or zero, but also more variable with the only significant difference between habitat types observed in the fall during daytime (Figure 5a,b). At the lower estuary location, net daily N_2_ exchange did not differ by habitat type and was at or near zero, suggesting a balance between diel periods in both summer and fall (Figure 4d).

Inorganic N exchange at the lower estuary location (SR) was dominated by ammonium as NO_x_ concentration was below analytical detection limits too often to establish a rate of change. Ammonium exchange showed significant differences between habitat types in the summer for both diel periods with ammonium exchange more negative (uptake) in SAV compared with adjacent BS (Figure 5c,d). The net daily exchange budget for ammonium at the SR location was also negative for SAV in the summer but near zero in BS (Figure 4e).

### Net oxygen exchange

**FIGURE 6:**
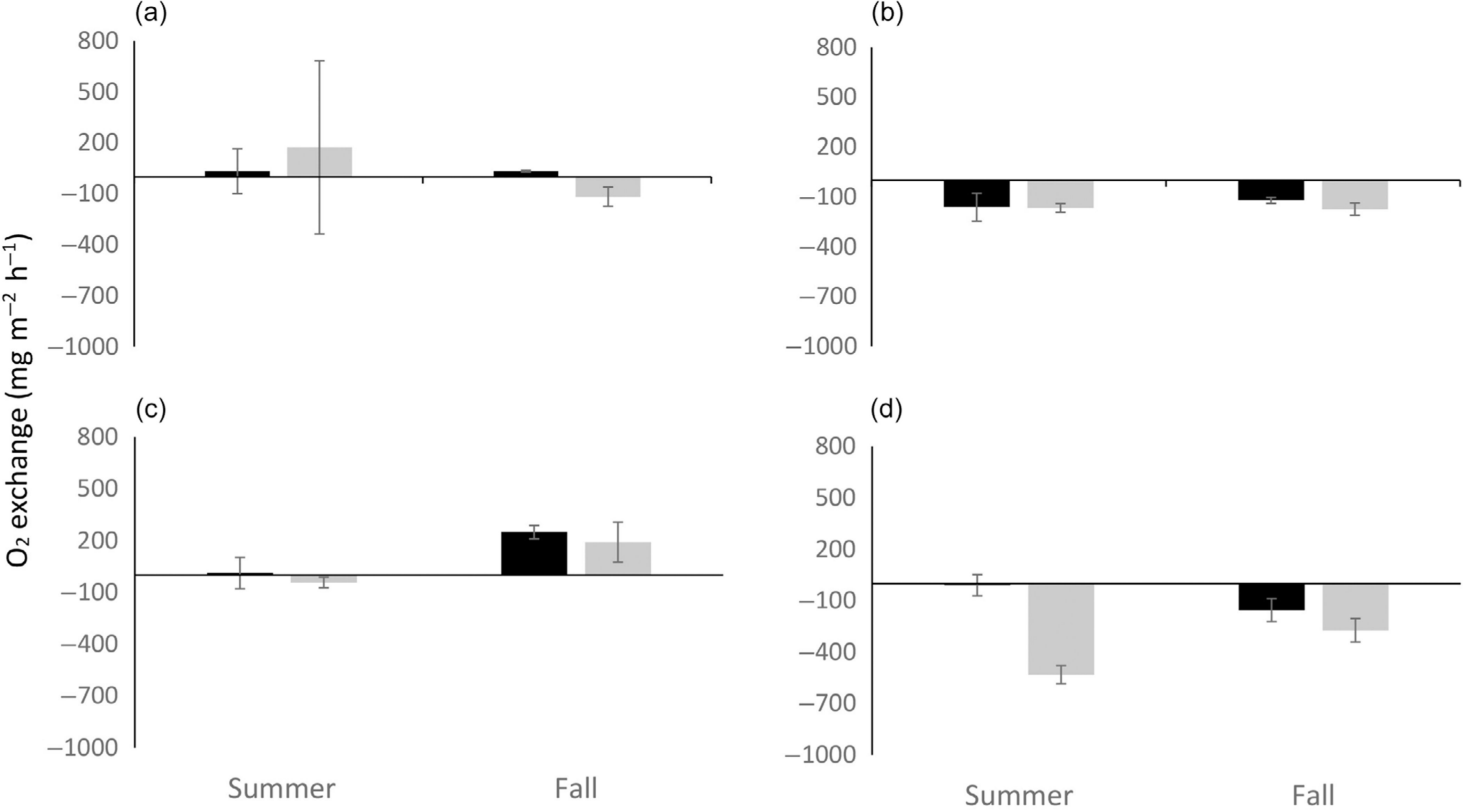
Mean (SD) net hourly exchange in dissolved O_2_ in the water column observed in in situ chamber incubations. Data are separated by Escambia River (a, b) and Santa Rosa Sound (c, d) and sample collection during the day (a, c) and at night (b, d). Bars within each figure are separated by season and habitat type: submerged aquatic vegetation (gray) and bare sediment (black). Incubations were 4–6 h in length. See text for further details.

Mean oxygen exchange was predictably positive or net zero during the day and negative or zero at night in both locations and seasons (Figure 6). No significant differences were detected between habitat types. Chamber DO concentrations were monitored to exclude hypoxic experimental trials (<20% saturation at any time during a trial) and did not have to exclude any replicates for this reason. Hypoxic conditions were observed in the summer at the ER location during long-term monitoring but not during our trial periods.

### Sediment content

Sediment analysis indicated that SAV and adjacent BS differ in total percent organic matter, as well as total nitrogen and carbon content, although habitat differences in sediment nitrogen changed between locations. Total percent organics and concentrations of carbon were consistently higher in SAV than in adjacent BS habitat at both locations (all *p* values <0.01). Total sediment nitrogen differed between habitats only at the SR location (*p* = 0.016) and displayed no difference among habitat types at the ER location (Table 1). The river mouth (ER) location had higher concentrations of all three constituents when compared with the lower estuary (SR) location (ANOVA *F*_1,32_ = 27.6, *p* << 0.01).

### Microbial genomic composition

**FIGURE 7:**
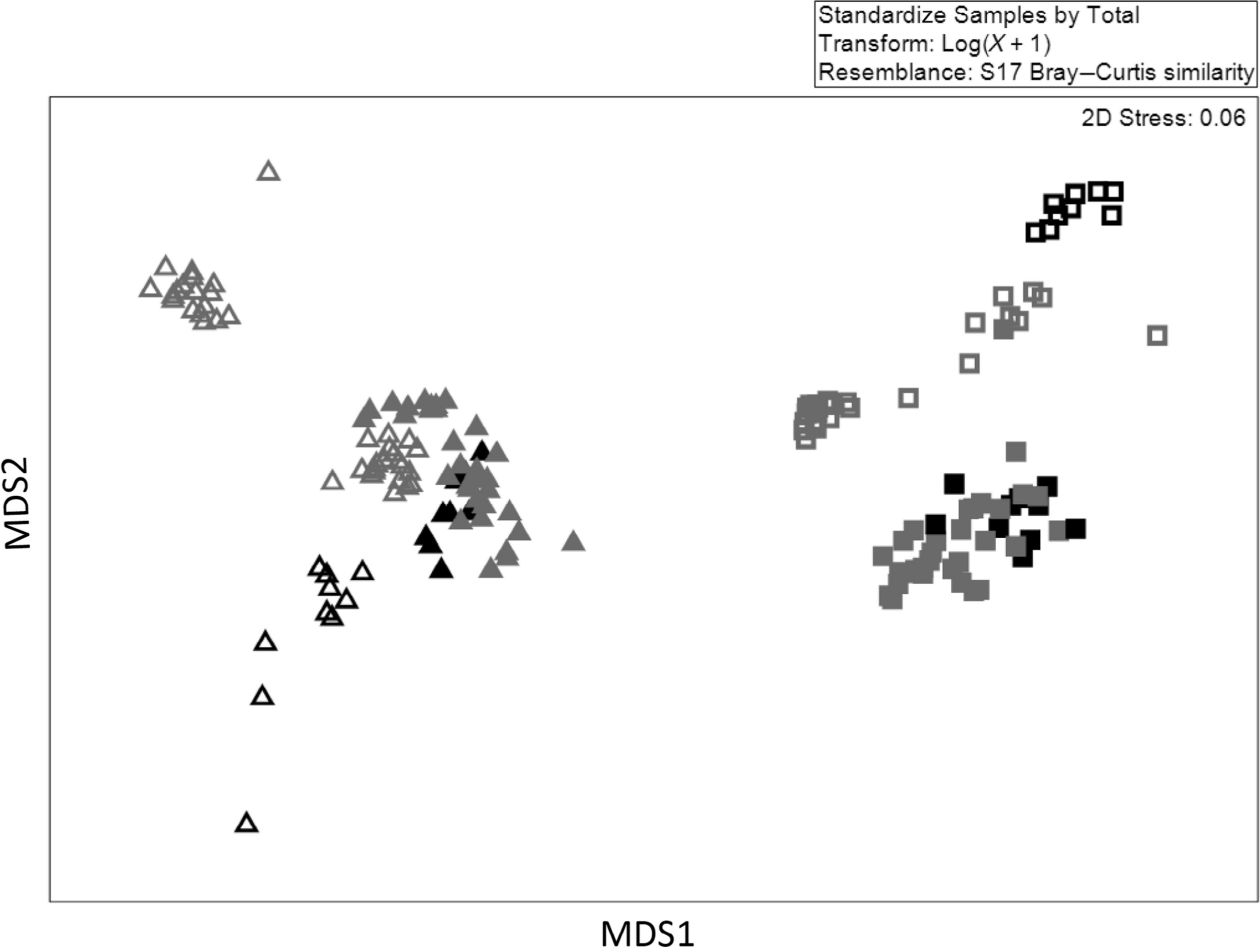
Multidimensional scaling (MDS) plot of 16S rRNA diversity differences based on Bray–Curtis similarity between seasons (summer, black; fall, gray) and habitat type (submerged aquatic vegetation, solid; bare sediment, open) within sample locations (Santa Rosa Sound, triangle; Escambia River, square).

Microbial genomic analysis was conducted on a filtered dataset containing 2,503,657 individual sequences (10,933 sequences/sample) representing 101,601 OTUs. Cluster analysis and ANOSIM showed significant differences among location/habitat type based on Bray–Curtis distances (*R* statistic 0.461; 0.1% significance; MDS, Figure 7). Microbial genetic differences between habitat types were larger and more consistent across seasons at the ER location than in SR. Microbial diversity in BS diverged from adjacent SAV habitat at the SR location, but those differences shifted between summer and fall. The most influential OTUs also differed between ER and SR with dissimilarity at ER locations based largely on OTUs from Orders Nostocales and Flavobacteriales more abundant in BS than in SAV habitat (Table 4). By contrast, SR locations were best delineated by higher abundance of OTUs from the Orders Bacteroidales and Desulfobacterales in SAV habitat (Table 4).

### The qPCR of targeted N-cycling genes

**FIGURE 8:**
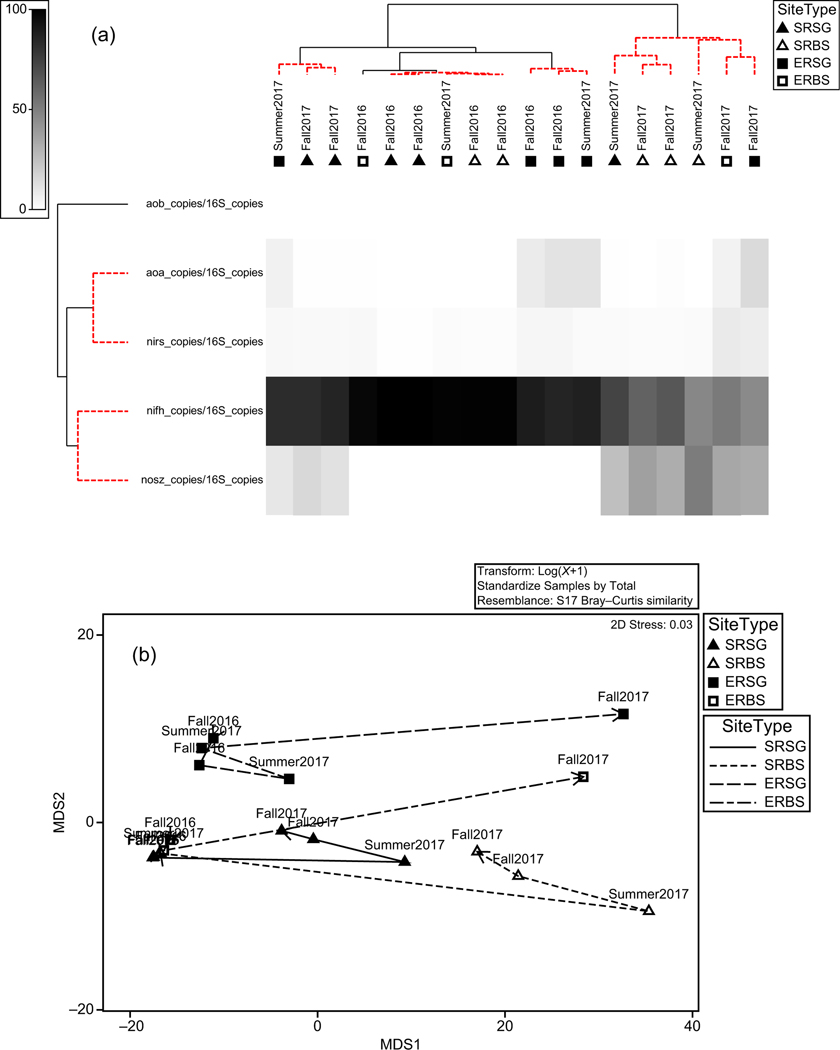
Shade plot (a) of functional gene copy number versus samples. Santa Rosa Sound (SR) and Escambia River (ER) samples were either bare sediment (BS) or submerged aquatic vegetation (SG). Multidimensional scaling (MDS) plot (b) of 16S-normalized copy number for select genes amplified with quantitative polymerase chain reaction based on Bray–Curtis similarity. Data are summarized for all genes combined. See Table 5 for details on all genes examined. Samples are summarized by symbol for location (SR, triangle or ER, square) and habitat type (SG, solid or BS, open) and labeled by season and year of collection.

Using Spearman ranks, the similarity matrix of the samples based on the nitrogen cycling gene copy numbers did not correlate with that based on the 16S rRNA gene (rho = 0.085, significance = 16.2%). PERMANOVA results for all genes combined were significant for all factors and combinations of factors. Variance explained was highest for Location, Season/Year followed by Location/Habitat type interaction (Table 5, Figure 8a,b). A clearer understanding of correlation between community structure and function is better investigated at the individual gene level. Our overall focus is on variation associated with habitat type within location as indicated by the Location × Habitat type factor in this study. Across all results for individual genes, variation associated with Location × Habitat type was significant for all genes examined except for *nirS*. Among the significant results, variance explained by this factor was highest for *amoA* followed by *nosZ* and lowest for the *nifH* gene (Table 5).

#### amoA

Using Spearman ranks, the similarity matrix of the samples based on the archaeal ammonium monooxygenase gene copy numbers correlated with that based on the 16S rRNA gene (rho = 0.532, significance = 0.1%). This indicates that community structure has some correlation with the presence of the *amoA* (archaeal), indicating that there is less functional redundancy with this gene than others.

PERMANOVA results indicate that the largest component of variation in *amoA* copy number was for Location and Habitat type within Location. Percent variance explained by both Location and Location/Habitat type interaction is significant and larger than error (Table 5).

#### amoB

Using Spearman ranks, the similarity matrix of the samples based on the bacterial ammonium monooxygenase gene copy numbers is not significantly correlated with that based on the 16S rRNA gene (rho = 0.09, significance = 16%). PERMANOVA results indicate that the largest component of variation in *amoB* copy number was by Location and Location/Habitat type interaction, which were both significant, and variance explained was about equal across both variables except Location × Habitat type × SeasonYear (Table 5). The residual variation is much higher than any of the factors, indicating that the factors chosen do not capture other sources of variation between samples (Table 5).

#### nifH

Comparison of normalized copy number across genes indicates *nifH* was the most prevalent gene examined in our sediment samples. Using Spearman ranks, the similarity matrix of the samples based on the *nifH* gene copy numbers is not strongly correlated with that based on the 16S rRNA gene (rho = 0.151, significance = 6.4%). PERMANOVA results indicate that the largest single component of variation in *nifH* copy number is due to SeasonYear and Location × SeasonYear interaction, suggesting a primarily spatial difference for this gene. All combinations of factors were significant including Location × Habitat type, indicating a habitat type different within Location (Table 5).

#### nirS

Using Spearman ranks, the similarity matrix of the samples based on the *nirS* gene copy numbers does not correlate with that based on the 16S rRNA gene. This is not unexpected, as *nirS* is found in many disparate taxa. PERMANOVA results indicate that the Location and SeasonYear and factor combinations that include both are significant factors in samples based on *nirS* relative abundance. However, the residual variation is higher than any of the factors, suggesting there is more variability for this gene that is not captured with our design factors (Table 5).

#### nosZ

Using Spearman ranks, the similarity matrix of the samples based on the *nosZ* gene copy numbers does not correlate strongly with that based on the 16S rRNA gene (rho = 0.138, significance = 6.1%). This is a bit unexpected, as *nosZ* is not widely dispersed across taxa. PERMANOVA results indicate that *nosZ* relative abundance is significantly affected by all factors and combinations in the design. Residual variation is greater than all sources other than Location, Season/Year, and combinations thereof (Table 5).

## DISCUSSION

### Habitat differences in nutrient exchange rates

The goal of this analysis was to explore habitat differences at the sediment–water interface that are potentially important for predicting the impact of within-location habitat shifts on delivery of the ecosystem function of mediating nitrogen exchange. Eyre and Maher (2011) demonstrated the value of functional habitat mapping at the scale of the whole estuary as a method for understanding the functional impacts of habitat change, but also demonstrated the need for habitat-specific data to support it. Habitat differences in biologically mediated nitrogen exchange reflect differences in the balance between the availability of labile nitrogen for metabolic processes and the capacity of the system to process excess nitrogen either through denitrification or burial. We deliberately chose two locations that differ in physical conditions, but are important locations for SAV and nutrient management. The focus is on habitat differences within locations, as this is most relevant to a functional assessment of annual changes in SAV coverage. Comparing adjacent habitat types minimizes other ancillary differences that mediate nitrogen exchange, such as salinity, temperature, DO concentration, or sediment composition. Once these larger scale physical environmental differences are minimized, the role of structural habitat differences like SAV presence should become more evident. In our study, habitat differences in nitrogen exchange were stronger during the day than at night, and these habitat differences were more apparent at the river mouth location than in the lower estuary. The net daily nitrogen budget, which combined these diel differences within location, further demonstrated that net exchange of N_2_ and inorganic nitrogen differed more between adjacent habitat types at the river mouth location particularly in the fall, but net diel habitat differences were not evident in our data as variability was high.

Expectations for habitat differences in nitrogen exchange were driven by observed differences in environmental condition between SAV and adjacent BS (Table 1). The largest habitat differences in environmental condition within location were for DO at the river mouth location and sediment organic content at the lower estuary location. The observed differences in daily minimum DO between SAV and BS could result in differences in nitrogen cycling, if the water column DO drops low enough to interfere with sediment oxidative state; although this probably does happen in SAV at the river mouth, hypoxia was not observed during this study. The estuary site had larger differences in sediment organic content between SAV and BS, and this difference, combined with the low concentrations of inorganic nitrogen in the water column, was expected to lead to larger habitat differences in nitrogen exchange at this location. Our results suggest habitat differences in nitrogen flux were small relative to within-site variability but contrary to expectations: the habitat differences we observed were larger at the river mouth location.

Habitat differences at the river mouth may be associated with higher structural complexity of SAV at this location. At the river mouth location, we observed efflux of nitrogen consistent with the higher concentrations of ambient nitrogen and carbon, as well as the decreased salinity and increased organic material in the sediment (Cornwell et al., 2016; Juhl & Murrell, 2008). Net daily exchange was more positive (efflux) for N_2_ in BS than in adjacent SAV, but more negative or neutral for inorganic N in BS. This pattern suggests a higher rate of processing ambient N in BS than in SAV, which may result from better water flow across the sediment surface in BS habitat and is important for evaluating changes in habitat coverage at the river mouth location.

Lack of habitat differences in the lower estuary may be associated with the low concentration of organic N in the water column. Daily nitrogen exchange at the estuary location favored net nitrogen fixation. Although habitat differences were minimal for N_2_ exchange, we did see indications of net daily habitat differences for exchange of ammonium, which was more negative (uptake) in SAV, particularly in the summer. Exchange of both N_2_ and NH_4_^+^ at the estuary location is consistent in pattern with reported rates at other similar locations with lower availability of ambient nitrogen and carbon (Russell et al., 2016). Ammonium exchange (uptake or release) generally reflects secondary production, as well as coupling of secondary production with sediment nitrification (Weston et al., 2010), and the minimal habitat differences suggest SAV is not sufficiently different from adjacent BS habitat to generate a detectable difference in nitrogen exchange.

Previous work in coastal estuaries has highlighted differences in nitrogen processing associated with both sediment and water column differences in habitat. Nitrogen exchange differences are reported along salinity gradients (Cornwell et al., 2016) and in response to light availability at the sediment surface (Murrell et al., 2009). Light availability and salinity impact primary production, and this process is tightly linked to nitrogen cycling (Juhl & Murrell, 2008). These differences are also more aligned with location differences in this study than with different adjacent habitat types within each of these locations. Differences in nitrogen cycling rates have also been reported for structural habitat such as emergent wetlands (Blackwell et al., 2010; Smyth et al., 2013) and sea grass (Carlson-Perret et al., 2019; Cole & McGlathery, 2012; Eyre & Ferguson, 2002). A comparison of our chamber-derived estimates of exchange for N_2_ and NO_x_ suggests that our rate estimates are similar but somewhat larger in magnitude and favor net nitrogen fixation compared with those reported elsewhere (Table 6). In a review of nutrient flux experiments, Boynton et al. (2018) noted flux estimates from in situ chambers have tended to be greater in magnitude compared with core estimates, and this has been attributed to reductions in soil compression, particularly in vegetated habitat. Our use of larger chambers and direct estimation of N_2_ based on MIMS minimize these effects, but also allows for a biased estimate caused by air bubbles in the experimental system (Eyre et al., 2002). Absence of bubbles was verified at each sampling event and steps were taken to reduce the likelihood of this issue, so we do not feel this is an important source of bias in this study. Rather, the differences in magnitude may simply reflect the value of an in situ technique that includes realistic flow dynamics and both sediment and water column processes in the resulting integrated flux estimate.

Previous research has also found important differences between SAV and adjacent BS under ambient conditions, but the results have been mixed. Zarnoch et al. (2017) reported net nitrogen fixation in eelgrass beds in New York State, but net denitrification when the system was enriched with NO_3_. These responses were more pronounced in SAV, probably due to the higher supply of organic carbon. These conditions are a mix of our two sample locations with higher salinity but also higher concentrations of inorganic N. At our N-limited location in the lower estuary, the main source for labile N may be nitrogen fixation, but while adjacent habitat did differ in sediment organic content in the lower estuary, this difference did not result in different nitrogen processing rates in SAV. In a similar study in Virginia, Aoki and McGlathery (2018) reported higher rates of denitrification in SAV compared with adjacent BS and reported “hotspots” in SAV that could be an order of magnitude higher than median reported rates. Finally, in a whole-system study based on similar methods, Eyre et al. (2011) reported higher rates of denitrification in SAV compared with all other habitats examined. However, the systems in Virginia and Australia were not reported to be N-limited. Russell et al. (2016) in an examination of estuarine sediment N flux in Australia reported that nitrogen fixation was the dominant process in intertidal *Zostera muelleri* beds, but not significantly different between SAV and adjacent BS, and this is most consistent with our observations of low habitat differences at the lower estuary location. They also reported that nitrogen fixation was terminated experimentally by N additions and stimulated by C additions. A higher rate of denitrification has been reported in estuaries elsewhere, particularly for vegetated habitats. However, evidence also points to a pattern of nitrogen fixation in estuarine vegetated sediment when labile nitrogen is limiting (Fulweiler et al., 2007; Herbert, 1999; Newell et al., 2016). We deliberately used an in situ approach and a chamber design with a larger sediment surface area (0.28 m^2^) for this study in order to observe sediment net nutrient exchange at a spatial scale closer to that of observed habitat shifts (~1 m year^−1^). The observed low habitat differences and the observation of net N-fixation at the lower estuary location are likely a result of both low ambient availability of nitrogen and the larger scale of the chamber design, which is more closely aligned with the central question of understanding realized habitat differences. The most important habitat differences may be dependent on the state of nitrogen loading in the system. An additional question is whether these state-dependent observations for nitrogen cycling are consistent with potential for nitrogen cycling reflected in the habitat-specific microbial communities.

### Microbial mediation of habitat differences

The change in the net nutrient exchange between habitat types in this study may reflect differences in sediment organic matter, as well as differences in the bacterial community that mediates nitrogen processing and bio-availability. Sediment total organic matter, organic carbon, and nitrogen were all higher in SAV compared with adjacent BS, but the habitat differences were larger at the lower estuary location. Microbial community composition also showed habitat differences at both locations, although microbial differences between habitat types within location were more seasonally consistent at the river mouth. This consistency is aligned with the larger habitat-specific differences in nitrogen exchange observed at the river mouth. A study of global patterns in microbial community structure among habitat reported the most important driver of difference was salinity (Lozupone & Knight, 2007), and this is consistent with comparisons of river mouth to lower estuary, but not with habitat differences within location observed at both locations. Seasonal clustering of the microbial community, particularly in BS in the lower estuary, was observed but did not diminish differences between SAV and adjacent BS. The BS examined was adjacent to SAV by design, which suggests smaller scale microbial community differences associated with habitat are important.

It is difficult to interpret the relative abundance of specific taxonomic groups for differences between habitats, particularly for nitrogen processing, which is ubiquitous across microbial orders. Nonetheless, examination of microbial community structure indicates potentially important differences between SAV and adjacent BS habitat. Previous studies of microbial community structure in SAV have found similar habitat differences (Devereux, 2005; Ettinger et al., 2017; James et al., 2006) and highlighted the higher prevalence of sulfate-reducing bacterial species (Devereux et al., 2011) as key mediators of exchange associated with the rhizosphere. We found similar patterns in this study with Family Desulfobacteracae, which was more common in SAV and the leading mediator of taxonomic difference between habitats in the lower estuary. A more diverse genomic community structure should lead to a community more responsive to variability in nitrogen loading and differences in microbial community structure between habitat types switched between the river mouth and lower estuary locations.

Examination of individual functional gene prevalence between habitats within location also suggests potentially important habitat differences. Individual gene copy number was somewhat correlated with 16S community structure differences suggesting functional differences for N-cycling were present. The most important differences were between seasons and between lower estuary and river mouth locations, which is consistent with differences in SAV species, salinity, sediment organic content, and incidence of hypoxia. All factors were known to influence N-cycling at the sediment–water interface. Differences in gene copy number among habitat types within locations were smaller, but significant, and this is consistent with observed differences in N-cycling rates among habitat types. In particular, the differences in *nifH* were observable between SAV and adjacent BS at both locations and in higher gene copy prevalence of the *nifH* gene in the lower estuary. In addition, the overall copy number for *nifH* was highest among all genes examined in this study and supports the chamber data as an indicator of the potential importance of nitrogen fixation at locations where N is limiting. This functional difference supports the conclusion that habitat-specific differences were small but detectable independent of more predictable location differences, and nitrogen fixation is potentially most important to nitrogen processing in the lower estuary.

### Habitat-specific ecosystem services and management

Overall, habitat appears to influence nitrogen processing more significantly in the lower salinity, more productive river mouth than in the higher salinity, and less organic-rich lower estuary where adjacent habitat differences appear less important. This conclusion is supported by both our chamber-derived budget for net nitrogen exchange and habitat-specific functional differences in the microbial community, although the prevalence of the *nifH* gene in SAV suggests potential for N-fixation in this habitat type is high. SR is a region of PBS where SAV has undergone documented expansion in coverage over the last 5–10 years. By contrast, SAV coverage near the river mouth location has declined steadily since the 1950s and represents a shift down estuary from historic SAV coverage in PBS (Lewis et al., 2008). The functional importance of this habitat shift to overall delivery of ecosystem services within PBS has not been evaluated, but the findings here suggest it is important at least for the service of processing excess nitrogen. Healthy SAV habitat provides a suite of ecosystem services besides processing of anthropogenic nitrogen (Lewis et al., 2016). Yet, setting nitrogen loading criteria for PBS should account for natural processing of nitrogen so that improvement in coverage of habitat is most likely to be associated with an increase in ecosystem function and may allow for increased allowable loading. Understanding small-scale habitat differences, such as conversion of BS to SAV, and scaling these differences up to the whole estuary are critical components of assessment from an ecosystem services prospective. Understanding the differences in services provided by habitat types can inform decisions about habitat loss or restoration, as well as decisions about nitrogen loading in the context of the ecosystem’s ability to process those nutrients naturally. These data will be incorporated into estuarine habitat maps and models to inform and improve decision-making of habitat change for improving ecosystem services in estuaries.

## Figures and Tables

**TABLE 1:** Summary data for two study locations, Escambia River (ER) and Santa Rosa Sound (SR), and two shallow-water habitat types (submerged aquatic vegetation [SAV] and adjacent bare sediment [BS]) in Pensacola Bay System, FL, USA.

Habitat type by location	Mean salinity	Mean water temperature (°C)	DO (mg L^−1^)	Percent organic content	Organic C (mg g sed^−1^)	Total N (mg g sed^−1^)	Vegetation spp.	NH_4_ (μmol L^−1^)	NO_x_ (μmol L^−1^)
ER	5							1.27 (1.0)	5.9 (4.3)
SAV		26.1	3.5 (1.9)	1.04 (0.2)	3.28 (3.1)	0.22 (0.25)	*Ruppia* spp./*Vallisneria* spp.		
BS		26.9	6.0 (4.9)	0.63 (0.2)	2.34 (1.1)	0.21 (0.12)			
SR	22							0.36 (0.43)	0.22 (0.19)^a^
SAV		24.8	6.2 (4.7)	0.83 (0.2)	3.1 (1.8)	0.34 (0.24)	*Thalassia* spp./*Halodule* spp.		
BS		24.9	7.8 (6.5)	0.23 (0.02)	2.35 (1.1)	0.05 (0.05)			

*Note*: Dissolved oxygen (DO) values are mean and minimum hourly values in parentheses. Sediment (sed) organic content (in percentage), total organic carbon, total nitrogen, and nitrogen concentration in site water samples are given as mean with SD in parentheses.

a Value given for NO_x_ excludes <minimum detectiuon limit (MDL) results.

**TABLE 2:** Primer details for quantitative polymerase chain reaction analysis of target genes.

Gene target	Primer name	Primer
*nirS*	nirSCd3aF nirSR3cd	AACGYSAAGGARACSGG GASTTCGGRTGSGTCTTSAYGAA
*nosZ* clade I	nosZ1F nosZ1R	WCSYTGTTCMTCGACAGCCAG ATGTCGATCARCTGVKCRTTYTC
*nifH*	IGK3 DVV	GCI WTH TAY GGI AAR GGI GGI ATH GGI AA ATI GCR AAI CCI CCR CAI ACI ACR TC
Archaeal *amoA*	GenAOAF GenAOAR	ATAGAGCCTCAAGTAGGAAAGTTCTA CCAAGCGGCCATCCAGCTGTATGTCC
Bacterial *amoA*	amoA-1Fmod GenAOBR	CTGGGGTTTCTACTGGTGGTC GCAGTGATCATCCAGTTGCG
16S	515f 806r	GTGYCAGCMGCCGCGGTAA GGACTACNVGGGTWTCTAAT

**TABLE 3:** Summary of gBlock used for quantitative polymerase chain reaction expression of target genes.

Gene target	Size (bp)	Organism	Accession
*nirS*	480	*Pseudomonas stutzeri*	X53676
*NirK*	179	*Sinorhizobium meliloti*	AE006469
*nosZ I*	380	*Pseudomonas fluorescens*	AF197468
*nifH*	470	*Azotobacter vinelandii*	M20568
Archeal *amoA*	201	*Candidatus nitrosphaera*	CP007174
Bacterial *amoA* a	180	*Nitrosomonas europaea*	L08050
Bacterial *amoA* b	207	*Nitrosospira briensis*	U76553

*Note*: Bacterial *amoA* a and b were used in a 50:50 volume ratio as standard.

**TABLE 4:** Most influential operational taxonomic units (OTUs) separating submerged aquatic vegetation (SAV) and adjacent bare sediment habitats based on analysis of similarity of genomic data. Relative abundance values are based on Bray–Curtis distances and influence cutoff is 40% of cumulative contribution curve for all OTUs analyzed at the family taxonomic level.

	Mean abundance		
Family by location	SAV	Bare sediment	Relative contribution (%)
Santa Rosa Sound			
Desulfobacteraceae	35.23	26.72	8.64
Bacteroidetes_BD2-2	20.21	11.44	8.22
Flavobacteriaceae	18.48	21.62	5.51
Bacteroidales_unclassified_family	8.79	3.86	5.21
Gammaproteobacteria_unclassified_order	26.83	25.73	4.24
Prolixibacteraceae	4.55	4.65	3.29
Woeseiaceae	10.7	13.62	3.1
Escambia River			
Nostocales_unclassified_family	3.62	11.49	7.41
Gammaproteobacteria_unclassified_order	4.36	10.19	5.34
Flavobacteriaceae	10.21	13.36	4.68
Bacteroidetes_BD2-2	6.64	8.26	3.44
Chromatiaceae	2.07	5.74	3.4
Woeseiaceae	8.25	7.13	3.22
Cyclobacteriaceae	5.7	7.24	3.22
Desulfobacteraceae	25.47	27.84	3.21
Burkholderiaceae	5.12	8.56	3.17

**TABLE 5:** Permutational multivariate analysis of variance results of 16S normalized gene copy number by treatment group.

Treatment	df	Overall	*amoA*	*amoB*	*nirS*	*nifH*	*nosZ*
Location	1	7.685**	41.727**	15.03**	11.316**	9.1394**	32.288**
Habitat type	1	6.8785**	17.981*	12.812**	−1.1171	5.1216**	12.296**
SeasonYear	2	16.408**	16.238	13.037**	6.5083**	22.356**	45.185**
**Location × Habitat type**	**1**	**8.7804** **	**34.801** *	**10.942** **	**−1.7148**	**9.2085** **	**20.222** **
Location × SeasonYear	2	18.343**	−7.9729	12.259**	4.379*	39.353**	34.813**
Habitat type × SeasonYear	2	3.4	8.0809	10.611**	1.5941	9.0226**	14.233**
Location × Habitat type × SeasonYear	2	6.6568*	−14.427	4.5313	6.6688*	19.553**	21.004**
Error	6	3.6144	30.71	25.724	13.306	17.363**	21.194**

*Note*: Values are estimated variance explained with significance value indicated by asterisks (*). Overall results are for all examined genes combined. Independent variables are sample Location (Escambia River/Santa Rosa Sound), Habitat type (submerged aquatic vegetation, bare sediment), and SeasonYear (summer/fall × 2016/2017). Bold type highlights primary comparison of habitat type within location.

* *p* ≤ 0.05;

** *p* ≤ 0.001.

**TABLE 6:** Comparison of nutrient flux values from other estuaries.

Study location	Habitat (spp.)	In situ/core	N_2_	NO_x_	NH_4_	Reference
Atchafalaya River, LA, USA	BS	Model	6.22	N/A	N/A	Bennett et al. (2016)
Big Lagoon, FL, USA	SAV (*Thalassia testudinum, Halodule beaudettei*)	Core	N/A	−26.6 to 26^a^	−37 to 22.7	Hester et al. (2016)
Chesapeake Bay	BS^b^	Core	50 to 600	2000	3510	Kellogg et al. (2013)
Escambia River, FL, USA	BS	In situ	−399.6	−228.9	104.7	This study
Escambia River, FL, USA	SAV (*Ruppia maritima*)	In situ	33.2	−30.3	102.7	This study
Moreton Bay, Australia	SAV (*Zostera* spp., *Halophila* spp.)	In situ	0 to 200	0 to 6	−60 to 90	Eyre et al. (2011)
Moreton Bay, Australia	BS	In situ	20 to 120	6 to 18	−100 to 200	Eyre et al. (2011)
Pensacola Bay, FL, USA	BS	Core	N/A	−44.2 to −9.6^c^	−40 to 27^c^	Smith and Caffrey (2009)
Santa Rosa Sound, FL, USA	BS	In situ	−679.8	N/A	20.6	This study
Santa Rosa Sound, FL, USA	SAV (*Thalassia* spp.)	In situ	−470.3	N/A	−38.4	This study
Revellata Bay, Spain	SAV (*Posidonia oceanica*)	Core	N/A	−20.6 to 4.1	−240 to 5.5	Gobert et al. (2002)
Ria Formaosa, Portugal	SAV (*Zostera noltii*)	Core	N/A	−63 to −30	−92 to 27	Asmus et al. (2000)

*Note*: Habitat is given for each study as bare sediment (BS) or submerged aquatic vegetation (SAV) with SAV species if reported. N/A indicates no reported value for that nutrient. Values are given either as a range or mean as reported in the cited reference.

a Data for NO_3_ only.

b Includes adjacent oyster bed.

c Data reported in millimoles per square meter per day and converted to micromoles per square meter per hour based on 24-h day.

## Data Availability

Data (Fulford, 2022) are available from the EPA Science Hub: https://doi.org/10.23719/1504273.
